# Ethological Constraints and Welfare-Related Bias in Laboratory Mice: Implications of Housing, Lighting, and Social Environment

**DOI:** 10.3390/ani16020314

**Published:** 2026-01-20

**Authors:** Henrietta Kinga Török, Boróka Bárdos

**Affiliations:** 1 Institute of Genetics and Biotechnology, Hungarian University of Agriculture and Life Sciences, 4 Szent-Györgyi A., 2100 Gödöllő, Hungary; kingahtorok@gmail.com; 2 Institute of Animal Sciences, Hungarian University of Agriculture and Life Sciences, 40 Guba S., 7400 Kaposvár, Hungary

**Keywords:** laboratory mouse welfare, domestication syndrome, ethological mismatch, environmental enrichment, circadian disruption

## Abstract

Laboratory mice are the most widely used model organisms in biomedical and behavioral research, yet growing concerns regarding reproducibility and translational validity have highlighted the substantial influence of housing and husbandry conditions on experimental outcomes. Although domestication is often assumed to have rendered laboratory mice fully adapted to artificial environments, evidence from ethology indicates that many core behavioral and physiological needs remain conserved. As a result, standard laboratory housing may generate chronic stress, alter behavior, and introduce systematic bias into experimental data. This narrative review critically examines how ethological constraints persisting after domestication interact with key environmental factors, social housing, environmental enrichment, ambient temperature, and lighting regimes to shape welfare and experimental validity in laboratory mice. Rather than providing an exhaustive overview of mouse behavior, the review adopts a problem-oriented and solution-focused approach, highlighting specific welfare-related mechanisms that can distort behavioral and physiological readouts. Particular attention is given to social isolation and aggression in male mice, the role of nesting material in mitigating thermal stress, and the effects of circadian disruption under standard and reversed light–dark cycles. By integrating ethological theory with laboratory animal welfare research, this review argues that housing conditions should be regarded as integral components of experimental design rather than secondary technical variables. Addressing welfare-related bias through evidence-based refinement strategies is essential for improving reproducibility, enhancing data interpretability, and strengthening the scientific validity of mouse-based research.

## Simple Summary

Laboratory mice are the most widely used animals in biomedical and behavioral research. Although they have been domesticated for many generations, they still retain many fundamental behavioral and physiological needs shaped by their evolutionary history. Standard laboratory housing often fails to meet these needs, which can negatively affect both animal welfare and the reliability of scientific results. This review summarizes evidence showing that common housing conditions such as social isolation, limited environmental complexity, low ambient temperature, and testing during the animals’ inactive phase can create chronic stress and alter baseline physiology and behavior in mice. These effects are often subtle but systematic, meaning they can bias experimental outcomes and contribute to poor reproducibility between studies. We highlight three key areas where welfare-related bias is particularly important: the social environment, access to nesting material and thermal regulation, and lighting regimes that disrupt circadian rhythms. Improving housing conditions in these domains is not only an ethical responsibility but also a practical strategy to enhance data quality and translational relevance. Treating housing and husbandry as integral parts of experimental design can therefore benefit both animal well-being and scientific rigor.

## 1. Introduction

The laboratory mouse (*Mus musculus*) occupies a central position in modern biomedical and behavioral research, with millions of individuals used annually across diverse experimental paradigms. Despite this extensive reliance, concerns regarding the reproducibility and translational validity of preclinical findings have intensified over the past two decades. Multiple analyses have demonstrated that a substantial proportion of experimental results cannot be reliably replicated or translated into clinical benefit, giving rise to what is now widely referred to as the reproducibility crisis in biomedical science [1,2,3,4,5].

Although this crisis is often attributed to methodological shortcomings, insufficient statistical power, or publication bias, an increasingly recognized yet less systematically addressed factor is the biological validity of the animal models themselves. Laboratory mice are typically housed in highly standardized but environmentally impoverished conditions that differ markedly from the ecological and social contexts in which their behavioral and physiological systems evolved. Such environments may constrain the expression of species-typical behaviors and generate systematic bias, rather than random experimental noise [6,7].

Domestication is frequently assumed often implicitly to have produced animals that are fully adapted to laboratory life. However, growing evidence suggests that domestication represents a mosaic process in which certain traits, such as fearfulness toward humans or stress hormone reactivity, are selectively modified, while core behavioral repertoires remain largely conserved [8,9]. As a result, laboratory mice continue to exhibit strong motivations related to nesting, social organization, environmental control, and circadian regulation, even after many generations of captive breeding.

Failure to accommodate these conserved ethological needs may lead to chronic, low-level stress that subtly but persistently alters baseline physiology and behavior. Such alterations can fundamentally affect experimental outcomes, particularly in behavioral neuroscience and psychopharmacology, where baseline emotional and motivational states play a decisive role [10,11]. Importantly, these effects often remain undetected by routine welfare assessments, yet they may systematically distort the interpretation of experimental data.

Recent critiques have further questioned whether commonly used behavioral paradigms accurately measure the constructs they are assumed to assess, especially when animals are tested under ethologically mismatched conditions [12,13]. From this perspective, poor reproducibility may arise not only from technical variability but from a deeper misalignment between experimental design and the biological characteristics of the model organism. Beyond experimental outcomes, long-term housing conditions have been shown to influence animal welfare in ways that may not be immediately detectable through standard health monitoring, yet still affect behavioral stability and data interpretation [14,15]. Recent meta-analyses have demonstrated that conventional laboratory housing not only increases morbidity and all-cause mortality in rodents [16], but also creates abnormal biological systems that lack ecological relevance, potentially hindering the translation of research findings to human health [17]. Therefore, integrating ethological knowledge into laboratory management is essential to improve both welfare and scientific outcomes.

In this review, we argue that addressing the reproducibility problem in mouse-based research requires explicit recognition of this ethological gap. Rather than providing an exhaustive catalogue of mouse behavior, we adopt a problem-oriented approach that examines how specific aspects of laboratory housing interact with conserved ethological traits to shape welfare and experimental validity. We focus on three domains where this interaction is particularly consequential: (1) social environment and the management of isolation and aggression; (2) environmental enrichment and thermal regulation, with an emphasis on nesting material as a biological necessity; and (3) lighting regimes and circadian alignment, including the implications of testing animals outside their natural active phase.

By integrating classical ethological insights with contemporary welfare science and reproducibility research, this review aims to demonstrate that housing conditions should be treated as integral components of experimental design rather than secondary technical variables. Improving biological relevance through evidence-based refinement is therefore not only an ethical responsibility but also a practical strategy to enhance data reliability and translational relevance. To facilitate implementation, a summary table outlining key ethological considerations, welfare implications, and experimental consequences is included at the end of the manuscript (Table 1).

## 2. Methodology

### 2.1. Literature Selection and Scope

This review is based on a structured, narrative synthesis of the scientific literature addressing laboratory mouse welfare, ethology, and housing-related sources of experimental bias. A total of approximately 91 peer-reviewed publications were integrated in detail, covering classical ethology, laboratory animal welfare, behavioral neuroscience, physiology, and reproducibility research. These works were selected based on their relevance to the core themes of the review.

The scope of the review was intentionally focused on factors that are both ethologically grounded and experimentally consequential. Specifically, the literature was selected if it addressed (i) conserved behavioral traits persisting after domestication, (ii) social organization, isolation, and aggression in laboratory mice, (iii) environmental enrichment, nesting behavior, and thermal physiology, or (iv) lighting regimes and circadian organization. Priority was given to studies demonstrating clear links between housing conditions, welfare outcomes, and changes in behavioral or physiological baseline states.

### 2.2. Inclusion Criteria

Publications were included if they met at least one of the following criteria:

1. Empirical studies examining the behavioral, physiological, or neurobiological effects of housing, social environment, temperature, enrichment, or lighting in laboratory mice.
2. Comparative or ethological studies of wild, feral, or free-living mice that provided insight into conserved behavioral needs relevant to laboratory conditions.
3. Review articles, theoretical papers, or methodological analyses addressing animal welfare, reproducibility, or experimental bias in preclinical research.
4. Regulatory or guideline-oriented publications relevant to laboratory animal housing and refinement strategies.

Both classical foundational works and contemporary studies were included to ensure continuity between historical ethological insights and modern laboratory practice.

### 2.3. Exclusion Criteria

Publications were excluded if they focused exclusively on technical biomedical outcomes without reference to housing or welfare-related variables, or if their relevance to mouse ethology and laboratory conditions was indirect or purely speculative. Non-peer-reviewed sources were used only when widely recognized as authoritative (e.g., seminal books or guideline documents).

### 2.4. Synthesis Approach

Rather than applying a formal meta-analytic framework, this review employed a critical narrative synthesis. Studies were grouped thematically and evaluated in relation to their contribution to understanding welfare-related bias and ethological mismatch in laboratory mice. Particular emphasis was placed on identifying mechanistic links between housing conditions and altered baseline phenotypes, as these mechanisms are central to reproducibility and translational validity.

Conflicting findings were not excluded but were discussed in the context of strain differences, housing configurations, developmental timing, and methodological variability. This approach allowed for a nuanced interpretation of heterogeneous results and avoided oversimplification of complex welfare–science interactions.

### 2.5. Limitations

As a narrative review, this synthesis does not aim to provide exhaustive quantitative comparisons or effect size estimates. Instead, its strength lies in integrating diverse lines of evidence into a coherent conceptual framework. While the literature selection was systematic within the predefined thematic boundaries, some degree of selection bias is inherent to narrative approaches. However, by grounding the analysis in a well-defined reference corpus and prioritizing studies with clear ethological and methodological relevance, the review seeks to provide a balanced and scientifically robust overview.

## 3. Results and Discussion

### 3.1. Evolutionary and Ethological Constraints Persisting After Domestication

Domestication is often implicitly treated in laboratory practice as a process that renders animals fully adapted to artificial environments. In the case of the laboratory mouse (*Mus musculus*), this assumption underpins many standardized housing and husbandry protocols, which presume that long-term captive breeding has eliminated or substantially reduced ancestral behavioral needs. However, both classical ethological theory and contemporary welfare science indicate that domestication is not a comprehensive transformation, but rather a mosaic process in which some traits are selectively modified while others remain fundamentally conserved [8,18,19].

#### 3.1.1. Domestication as a Mosaic Process

Early evolutionary perspectives already emphasized that domestication primarily affects traits under direct or indirect human selection, such as docility, reproductive output, or morphology, while leaving much of the species-typical behavioral repertoire intact [18,19]. More recent syntheses reinforce this view, describing domestication as a syndrome involving correlated changes in stress physiology, neuroendocrine regulation, and morphology, without implying the loss of behavioral complexity or motivational systems [20,21].

In laboratory mice, selective breeding has reduced overt fear responses toward humans and altered hypothalamic–pituitary–adrenal axis reactivity in many strains. Nevertheless, core motivations related to exploration, nesting, social interaction, and environmental control remain robustly expressed across strains and experimental contexts [8,22,23,24]. Consequently, laboratory mice remain biologically prepared to respond to environmental and social cues that are often absent, distorted, or uncontrollable under standard housing conditions.

Importantly, domestication effects are strongly strain dependent. Inbred strains differ markedly in anxiety-like behavior, aggression, stress responsiveness, and coping strategies, reflecting both their mixed subspecies origins and divergent selection histories [25,26,27]. Treating laboratory mice as a behaviorally uniform population therefore obscures biologically meaningful variation and may contribute to inconsistent experimental outcomes across laboratories.

#### 3.1.2. Insights from Wild and Free-Living Mice

Comparative studies of wild, feral, and free-living *M. musculus* populations provide essential insight into which behavioral traits persist despite domestication. Field observations consistently document complex spatial organization, territoriality, dominance hierarchies, and extensive use of shelters and nests [22,28,29,30]. These features reflect core regulatory mechanisms governing stress, reproduction, and social stability rather than optional ecological embellishments.

Even under commensal conditions, wild house mice actively construct nests, regulate microclimates, and manage social stress through spatial avoidance or dispersal [24,31]. Laboratory housing, by contrast, restricts these regulatory options through spatial confinement and enforced proximity, forcing animals to cope with social and environmental challenges in fundamentally altered ways.

Comparisons with closely related species further underscore the persistence of ancestral ethological constraints. The mound-building mouse (*Mus spicilegus*), for example, exhibits highly specialized communal and architectural behaviors adapted to seasonal environmental stressors [32,33]. Despite ecological divergence, both *M. spicilegus* and *M. musculus* share fundamental needs for shelter, social regulation, and nocturnal activity patterns [34,35], highlighting the deep evolutionary roots of these traits.

Field studies further demonstrate substantial individual and population-level variation in aggressiveness and social tolerance, emphasizing that social behavior in mice is flexible and context dependent rather than uniform [36].

#### 3.1.3. Ethological Mismatch and Welfare-Related Bias

The discrepancy between conserved behavioral motivations and restricted laboratory environments can be conceptualized as an ethological mismatch. Rather than producing acute distress, such mismatch often manifests as chronic, low-level stress that alters baseline physiology and behavior over extended periods [8,37]. These effects may remain undetected by routine health monitoring yet exert profound influence on experimental outcomes.

Crucially, welfare-related bias arising from ethological mismatch is not random. Housing-induced alterations in metabolism, immune function, and emotional reactivity may systematically shift baseline phenotypes, thereby confounding experimental manipulations [5,6,7]. From this perspective, biologically impoverished environments do not simply increase variability but may actively generate structured bias that undermines reproducibility.

Recent methodological critiques have emphasized that behavioral assays conducted under ethologically inappropriate conditions may fail to measure the constructs they are intended to assess [9,13]. If baseline motivational and emotional states are altered by housing constraints, then experimental outcomes may reflect these underlying distortions rather than the effects of experimental variables of interest.

#### 3.1.4. Implications for Welfare and Experimental Design

Recognizing the persistence of ethological constraints after domestication has direct implications for both animal welfare and scientific rigor. Behaviors such as nest building, social interaction, and environmental manipulation should be regarded as biologically grounded needs rather than optional enrichments [38,39]. When these needs are chronically frustrated, animals may exhibit compensatory behaviors or altered physiological states that compromise the interpretability of experimental data.

From an experimental design perspective, failure to account for ethological mismatch risks conflating treatment effects with housing-induced artifacts. Addressing these constraints through evidence-based refinement therefore represents not merely an ethical improvement but a methodological necessity for enhancing reproducibility and translational relevance [3,4].

### 3.2. Social Environment, Isolation, and Welfare-Related Bias

The social environment constitutes one of the most powerful yet experimentally underappreciated determinants of mouse welfare and baseline phenotype. In free-living populations, social interactions regulate access to resources, mating opportunities, and shelter, while also shaping stress responsiveness and behavioral development [22,28,29]. Laboratory housing fundamentally alters these regulatory mechanisms by constraining space, limiting social choice, and enforcing artificial group compositions, thereby transforming social stress from a situational challenge into a potentially chronic condition.

#### 3.2.1. Natural Social Organization and Olfactory Regulation

House mice are highly social animals whose interactions are structured through dominance hierarchies, kinship, and territorial boundaries. These systems are dynamic and self-regulating: subordinate individuals can avoid dominant conspecifics through dispersal or spatial segregation, and social instability is often resolved by emigration rather than prolonged conflict [24,36]. Olfactory communication plays a central role in maintaining this balance. Urinary scent marks convey information about individual identity, dominance status, and reproductive condition, allowing mice to anticipate and avoid aggressive encounters [40,41].

Standard laboratory husbandry practices, particularly frequent cage cleaning, repeatedly disrupt these olfactory landscapes. Removal of scent marks destabilizes established social relationships and may increase aggression and anxiety-like behavior, even in otherwise stable groups [42,43]. Such disruptions are rarely acknowledged as experimental variables, despite their clear relevance for welfare and data interpretation. For this reason, it is recommended to always transfer mice to a clean cage using a portion of their used but clean nesting material to preserve olfactory cues [44].

#### 3.2.2. Social Isolation as a Chronic Stressor

Despite the social nature of mice, individual housing is commonly employed as a preventive measure against aggression, especially in adult males. However, a substantial body of evidence indicates that social isolation constitutes a potent and biologically meaningful stressor. Isolated mice exhibit increased anxiety-like and depressive-like behaviors, altered reward sensitivity, cognitive impairments, and dysregulated hypothalamic–pituitary–adrenal axis activity [11,45,46].

At the neurobiological level, social isolation induces long-lasting alterations in synaptic plasticity, neurotransmitter systems, and gene expression patterns associated with emotional regulation and social behavior [10,47]. Importantly, these changes may persist beyond the isolation period, effectively redefining the baseline phenotype of the animal. From an experimental standpoint, this raises a critical concern: treatment effects observed in isolated mice may reflect interactions with an isolation-induced phenotype rather than responses to the experimental manipulation itself [48].

#### 3.2.3. Aggression and Chronic Social Stress in Group-Housed Males

Group housing is often presented as a welfare-friendly alternative to isolation, yet this assumption requires careful qualification in the context of male mice. Under laboratory conditions, male mice frequently exhibit elevated levels of aggression, leading to injury, wounding, or mortality [23,49]. Unlike in natural settings, subordinate individuals cannot escape or establish alternative territories, resulting in prolonged exposure to social stress.

Chronic social stress has been shown to affect immune function, metabolic regulation, and emotional behavior outcomes that are central to many biomedical research domains [50,51]. Moreover, aggression levels vary substantially across strains, age groups, and housing configurations, introducing additional variability that is rarely controlled or reported [25,52]. Chronic social stress has also been linked to immune dysregulation and altered host–microbiota interactions, suggesting that social housing conditions may indirectly affect disease-related outcomes in experimental models [50].

#### 3.2.4. Social Environment and Reproducibility

From a methodological perspective, social environment represents a major but frequently overlooked source of experimental variability. Differences in group composition, dominance stability, isolation history, and husbandry practices can generate divergent baseline states even within nominally identical experimental conditions [9,53]. Such variability directly undermines reproducibility by inflating within-group variance and obscuring true treatment effects.

Importantly, social stress does not merely introduce random noise. Instead, it may systematically bias results by shifting behavioral and physiological baselines in predictable directions, such as heightened anxiety or altered immune responsiveness [5,6]. Failure to account for these effects risks misattributing housing-induced phenotypes to genetic, pharmacological, or environmental manipulations.

#### 3.2.5. Refinement Strategies and Conceptual Limitations

A range of refinement strategies has been proposed to mitigate the negative effects of social housing constraints. These include maintaining stable group compositions, minimizing disruptive cage cleaning, preserving olfactory cues, and introducing environmental complexity that allows partial avoidance or retreat [42,44,48,54]. Physical modifications such as perforated partitions may permit visual and olfactory contact without direct physical interaction, reducing the severity of aggression while preserving some degree of social stimulation.

However, no single housing strategy can fully replicate the regulatory flexibility of natural social systems. Strain differences, developmental history, and experimental context all modulate outcomes, underscoring the need to treat social environment as an explicit experimental variable rather than a fixed background condition [12]. Stable social groups in laboratory settings typically consist of 2 to 5 members [52,55]. To reduce the number of singly housed animals, strategies such as the use of cage dividers or social housing of compatible males should be prioritized [56,57]. Transparent reporting and justification of social housing decisions are therefore essential for both welfare assessment and scientific interpretation.

### 3.3. Environmental Enrichment, Nesting Behavior, and Thermal Biology

Environmental enrichment is widely promoted as a key component of laboratory animal welfare; however, its conceptualization and implementation in mouse housing remain inconsistent and, in many cases, biologically superficial. Enrichment is often reduced to the provision of objects or stimuli, rather than being evaluated in terms of its capacity to allow animals to express species-typical behaviors and exert control over their environment [8,38,58]. This distinction is critical, as biologically irrelevant enrichment may fail to mitigate, or may even obscure, underlying welfare deficits.

#### 3.3.1. Enrichment as Biological Relevance Rather than Stimulation

From an ethological perspective, enrichment should be defined by its functional relevance to the animal rather than by its novelty or complexity per se. In mice, meaningful enrichment includes opportunities to hide, manipulate materials, regulate social exposure, and construct nests [39,59]. Environments lacking these opportunities may act as chronic stressors, even in the absence of overt pathology, leading to altered emotional reactivity and behavioral rigidity [8,37].

Experimental studies demonstrate that environmental complexity enables “active coping” and behavioral regulation, allowing animals to respond to aversive stimuli through behavior rather than sustained physiological stress responses [60,61]. In contrast, barren cages restrict these regulatory strategies, increasing the risk of stereotypic behavior and long-term neurobehavioral changes that directly affect experimental readouts [8]. Importantly, the welfare consequences of barren housing may accumulate over time, influencing affective state and coping capacity even in the absence of overt pathology [14,15]. Recent evidence highlights that enrichment, particularly nesting material, is a biological necessity that directly influences thermal homeostasis and overall welfare [62].

#### 3.3.2. Ambient Temperature and Chronic Cold Stress

A central but frequently underestimated aspect of mouse housing is ambient temperature. Most animal facilities maintain temperatures between 20 and 24 °C, a range chosen primarily for human comfort rather than for the thermal biology of mice. This temperature range lies well below the thermoneutral zone of laboratory mice, which is estimated to be approximately 28–32 °C depending on strain, age, and activity level [63,64,65].

Housing mice under sub-thermoneutral conditions induces chronic cold stress, forcing animals to allocate metabolic resources toward heat production at the expense of growth, immune function, and disease resistance [51,66,67,68]. Importantly, this energetic burden does not necessarily manifest as overt distress, making cold stress an insidious but powerful source of welfare-related bias that can systematically affect experimental outcomes.

#### 3.3.3. Nesting Material as a Behavioral and Metabolic Necessity

Nest building is a highly conserved behavior in mice, serving critical functions in thermoregulation, reproduction, and psychological security. When provided with suitable nesting material, mice reliably construct nests that create warm, stable microclimates approaching thermoneutral conditions, even in relatively cold environments [54,69,70].

Numerous studies demonstrate that mice strongly prioritize access to nesting material over many other forms of enrichment, underscoring its fundamental biological importance [71,72]. Restricting nesting opportunities should therefore be regarded as a significant welfare deficit rather than a neutral housing condition. From an experimental perspective, lack of nesting material exacerbates strain- and sex-specific responses to cold stress, increasing variability and reducing reproducibility [66,73].

#### 3.3.4. Interactions Between Enrichment, Social Environment, and Space

The effects of enrichment cannot be evaluated independently of social housing and cage design. In group-housed mice, access to nesting material and shelters may be unevenly distributed due to dominance hierarchies, resulting in differential exposure to stress within the same cage [53,54]. Similarly, limited cage height and floor space may restrict the functional use of enrichment items, reducing their intended benefits [74,75].

These interactions complicate both welfare assessment and experimental interpretation. Enrichment strategies that improve outcomes in one strain or housing configuration may have neutral or adverse effects in another, contributing to inconsistent findings across laboratories [76]. Such context dependency highlights the importance of transparent reporting and justification of enrichment protocols.

#### 3.3.5. Enrichment, Standardization, and Reproducibility

A persistent concern in laboratory research is that environmental enrichment may increase variability and undermine experimental control. However, accumulating evidence challenges this assumption. Biologically impoverished environments may amplify variability by pushing animals into extreme or unstable physiological states, whereas controlled environmental complexity can stabilize baseline phenotypes [6,7,51].

This insight supports a shift away from rigid standardization toward biologically informed standardization, in which key ethological variables are systematically considered and documented [76]. Within this framework, enrichment, particularly nesting material, should be regarded as a tool for reducing welfare-related bias rather than as a confounding factor.

### 3.4. Lighting Conditions, Circadian Rhythms, and Measurement Validity

Lighting regimes represent one of the most pervasive yet systematically underestimated environmental variables in laboratory mouse research. As strictly nocturnal animals, mice possess sensory, physiological, and behavioral systems that are finely tuned to low-light environments and robust circadian organization. Standard laboratory lighting schedules, however, are typically designed around human working hours rather than the biological rhythms of the animals, resulting in a persistent temporal mismatch between experimental practice and endogenous circadian regulation [77,78].

#### 3.4.1. Circadian Organization and Behavioral Regulation

Circadian rhythms orchestrate a wide range of physiological and behavioral processes in mice, including activity patterns, hormone secretion, immune function, metabolism, and cognitive performance [78]. These rhythms are primarily entrained by light–dark cycles, with both light intensity and spectral composition playing critical roles in synchronization [79,80].

Disruption of circadian organization has been shown to induce profound and persistent changes in stress responsiveness, affective behavior, and disease susceptibility [81,82]. Importantly, circadian disruption may alter baseline phenotypes in ways that are not immediately apparent yet systematically influence experimental outcomes, particularly in behavioral and neurobiological studies.

#### 3.4.2. Light Intensity, Spectrum, and Welfare Implications

Standard laboratory illumination levels often exceed those encountered by mice during natural nocturnal activity. Such intensities may function as chronic stressors, especially for albino strains that lack retinal pigmentation and are therefore more vulnerable to photic damage [83,84].

Excessive or inappropriate lighting has been linked to retinal degeneration, altered sleep–wake cycles, endocrine disruption, and metabolic dysregulation [81,85]. Regarding light intensity, it is also recommended to maintain levels between 15 and 25 lux at the cage level to prevent retinal damage and minimize stress, particularly for albino strains, while following standardized measurement protocols [86]. This range is sufficient for routine husbandry while preserving circadian stability [87,88]. Despite these effects, lighting conditions are frequently underreported in experimental methods, limiting reproducibility and obscuring potential sources of between-study variability.

#### 3.4.3. Temporal Mismatch in Behavioral Testing

A major yet normalized source of bias in mouse research is the routine testing of animals during their inactive (light) phase. Many behavioral and physiological assays are conducted during daytime working hours, when mice would naturally be resting. This practice disregards circadian modulation of motivation, arousal, learning, and stress responses, leading to systematic distortions in experimental readouts [89].

Empirical studies demonstrate that testing during the inactive phase can yield qualitatively different results compared to testing during the active phase, affecting measures of anxiety-like behavior, social interaction, and cognitive performance [89,90]. Such differences raise fundamental questions regarding what is actually being measured under temporally mismatched conditions.

#### 3.4.4. Measurement Validity and the Reproducibility Debate

Recent methodological critiques have explicitly questioned whether commonly used behavioral paradigms in mice measure the constructs they are assumed to assess, particularly when experiments are conducted under ethologically inappropriate temporal conditions [9,13]. Circadian mismatch may alter motivational states and coping strategies, thereby changing task engagement rather than the targeted cognitive or emotional processes.

From this perspective, poor reproducibility may arise not only from technical variability but from systematic misalignment between experimental design and the biological characteristics of the model organism [1,4,5]. Addressing circadian validity is therefore central to improving both internal and external validity in mouse-based research.

#### 3.4.5. Reversed Light Cycles as a Refinement Strategy

Reversed light–dark cycles, in which the dark phase is shifted to coincide with human working hours, offer a potential solution to temporal mismatch by enabling testing during the animals’ natural active period. When implemented gradually and combined with appropriate control of light intensity and spectrum, reversed cycles have been shown to reduce stress responses and improve the reliability of behavioral assays [89,91].

Nevertheless, reversed light cycles are not a universal remedy. Inadequate acclimation, inappropriate light leakage during the dark phase, or failure to consider interactions with social and environmental factors may exacerbate circadian instability rather than alleviate it [13]. These limitations underscore the need for standardized reporting and evidence-based guidelines rather than ad hoc implementation.

#### 3.4.6. Implications for Experimental Design and Reporting

Lighting regimes and circadian timing should be treated as integral components of experimental design rather than as background conditions. Transparent reporting of photoperiod, light intensity, spectral composition, and timing of behavioral testing is essential for reproducibility and cross-study comparison [6,7,9].

Aligning experimental timing with the natural activity patterns of mice represents a biologically grounded refinement that benefits both animal welfare and data quality. More broadly, incorporating circadian considerations reinforces the central argument of this review: that improving biological relevance through ethologically informed housing and experimental design is a prerequisite for reliable and translatable science.

## 4. Conclusions

This review has argued that many of the limitations affecting the reproducibility and translational value of mouse-based research originate not solely from methodological or statistical shortcomings, but from a persistent mismatch between the ethological characteristics of mice and the standardized conditions under which they are housed and studied. Domestication and selective breeding have undoubtedly modified specific traits relevant to laboratory use; however, they have not eliminated core behavioral motivations related to social organization, environmental control, thermoregulation, and circadian regulation. Treating laboratory mice as if these motivations were irrelevant introduces systematic bias into experimental outcomes.

Across the domains examined, social environment, environmental enrichment and thermal biology, and lighting regimes, a recurring pattern emerges. Housing conditions that restrict the expression of species-typical behaviors do not merely reduce animal welfare in an ethical sense; they reshape baseline physiological and behavioral states in ways that directly affect experimental readouts. Social isolation and chronic aggression alter stress responsivity and neural plasticity; sub-thermoneutral housing conditions impose sustained metabolic costs; and circadian mismatch distorts motivation, cognition, and endocrine regulation. These effects are often subtle, chronic, and cumulative, making them difficult to detect through routine welfare assessments, yet highly consequential for scientific interpretation.

Importantly, welfare-related bias should not be conceptualized as random noise that can be averaged out through increased sample sizes or stricter standardization. Instead, the evidence reviewed here suggests that biologically impoverished environments may actively generate structured and directional bias, shifting baseline phenotypes in predictable ways. Such bias undermines reproducibility by increasing between-laboratory variability and complicating cross-study comparison, particularly in behavioral neuroscience, psychopharmacology, and immunology.

A key implication of this synthesis is that housing conditions must be recognized as integral components of experimental design rather than secondary technical details. Refinement strategies such as stable social grouping, preservation of olfactory cues, provision of adequate nesting material, and alignment of experimental timing with the animals’ circadian rhythms are not optional enhancements but scientifically justified interventions. When applied thoughtfully and reported transparently, these measures have the potential to reduce welfare-related bias, stabilize baseline phenotypes, and improve data reliability.

At the same time, this review does not advocate for uncritical complexity or the abandonment of experimental control. Rather, it supports a shift from rigid standardization toward biologically informed standardization, in which key ethological variables are systematically considered, justified, and documented. Such an approach aligns with emerging perspectives on controlled heterogenization and acknowledges that ecological validity and experimental rigor are not opposing goals but mutually reinforcing ones.

In conclusion, bridging the ethological gap between laboratory mice and their housing environments represents a powerful yet underutilized pathway toward improving both animal welfare and scientific quality. Addressing conserved behavioral needs through evidence-based housing and husbandry practices is not merely an ethical obligation but a methodological necessity. Future progress in mouse-based research will depend on the extent to which experimental design embraces this integrative perspective and treats the animal not as a passive tool, but as a biologically complex organism whose welfare and behavior fundamentally shape scientific outcomes.

## Figures and Tables

**Table 1:** Summary of evidence-based recommendations for laboratory mouse management.

Category	Mechanism/Issue	Welfare Impact	Effect on Data	Recommended Refinement
Social environment	Social isolation	Chronic stress; altered affective and neurobiological state	Biases behavioral baseline; shifts HPA axis	Group housing, compatible pairing, indirect social contact
Social environment	Male–male aggression	Social stress, wounding, mortality	Strain-dependent variability in immune/behavioral outcomes	Stable groups (2–5), avoid regrouping, use cage dividers
Olfactory environment	Scent cue disruption during cage cleaning	Anxiety; unstable hierarchies	Additional variance from social instability	Transfer part of used nesting material at cage change
Thermal biology	Sub-thermoneutral housing	Chronic cold stress; increased metabolic load	Alters metabolism, tumor growth, immune assays	Provide adequate nesting material; consider thermal needs
Enrichment/Nesting	Lack of nesting material	Impaired thermoregulation; increased stress	Inter-individual variance; metabolic confounds	Nesting material as standard provision
Lighting	High light intensity/unsuitable spectrum	Retinal/circadian stress (esp. albino strains)	Alters sleep, affect, metabolic readouts	Maintain ~15–25 lux at cage level
Circadian timing	Testing during inactive (light) phase	Reduced motivation; temporal mismatch	Systematic bias in behavioral tasks	Test during active (dark) phase or use reversed light cycle

## Data Availability

No new data were created or analyzed in this study. Therefore, data sharing is not applicable.
